# Supplementary material for: Microglial trogocytosis and the complement system regulate axonal pruning in vivo
Source: eLife. 2021 Mar 16;10:e62167. doi: 10.7554/eLife.62167 (PMC7963485; doi:10.7554/eLife.62167)
Supplement: Supplementary file 1. [file elife-62167-supp1.docx]

| **PLASMIDS USED OR GENERATED FOR THIS STUDY** | |  |  |
| --- | --- | --- | --- |
| Plasmid | Use | Source | Availability |
| pEGFP-N1 | Expression of eGFP in RGC axons | Clontech | Cat# 6085-1 |
| pFA6a pH-tdGFP | subcloning of pHtdGFP | Roberts et al., 2016 | Addgene plasmid 74322 |
| pEF1α P2A-pHtdGFP | Expression of pHtdGFP in RGC axons | This paper | Available from Edward Ruthazer upon request |
| SYP-GFP | subcloning of mouse SYP | Ruthazer et al., 2006 | Available from Edward Ruthazer upon request |
| pEF1α SYP-pHtdGFP | Expression of SYP-pHtdGFP in RGC axons | This paper | Available from Edward Ruthazer upon request |
| pEF1α aRCA3-Myc-P2A-pHtdGFP | Co-expression of aRCA3 and pHtdGFP in RGC axons | This paper | Available from Edward Ruthazer upon request |
| pEF1α aRCA3-mCherry-Myc-P2A-SYP-pHtdGFP | Co-expression of aRCA3-mCherry and SYP-pHtdGFP in RGC axons | This paper | Available from Edward Ruthazer upon request |
| pEF1α pHtdGFP-P2A-VAMP2 | Co-expression of pHtdGFP and VAMP2 in RGC axons | This paper | Available from Edward Ruthazer upon request |
| pEF1α pHtdGFP-P2A-Myc-VAMP2-C3 | Co-expression of pHtdGFP and VAMP2-C3 in RGC axons | This paper | Available from Edward Ruthazer upon request |
