# Supplementary material for: Microglial trogocytosis and the complement system regulate axonal pruning in vivo
Source: eLife. 2021 Mar 16;10:e62167. doi: 10.7554/eLife.62167 (PMC7963485; doi:10.7554/eLife.62167)
Supplement: Transparent reporting form [file elife-62167-transrepform.docx]

***eLife’s* transparent reporting form**

We encourage authors to provide detailed information *within their submission* to facilitate the interpretation and replication of experiments. Authors can upload supporting documentation to indicate the use of appropriate reporting guidelines for health-related research (see [EQUATOR Network](http://www.equator-network.org/%20)), life science research (see the [BioSharing Information Resource](https://biosharing.org/)), or the [ARRIVE guidelines](http://www.plosbiology.org/article/info:doi/10.1371/journal.pbio.1000412) for reporting work involving animal research. Where applicable, authors should refer to any relevant reporting standards documents in this form.

If you have any questions, please consult our Journal Policies and/or contact us: [editorial@elifesciences.org](mailto:editorial@elifesciences.org).

**Sample-size estimation**

- You should state whether an appropriate sample size was computed when the study was being designed
- You should state the statistical method of sample size computation and any required assumptions
- If no explicit power analysis was used, you should describe how you decided what sample (replicate) size (number) to use

Please outline where this information can be found within the submission (e.g., sections or figure legends), or explain why this information doesn’t apply to your submission:

Sample sizes were not precalculated using power analysis or other statistical frameworks. As this study uses new methods, it was not possible to calculate appropriate sample sizes during the design of the study.

**Replicates**

- You should report how often each experiment was performed
- You should include a definition of biological versus technical replication
- The data obtained should be provided and sufficient information should be provided to indicate the number of independent biological and/or technical replicates
- If you encountered any outliers, you should describe how these were handled
- Criteria for exclusion/inclusion of data should be clearly stated
- High-throughput sequence data should be uploaded before submission, with a private link for reviewers provided (these are available from both GEO and ArrayExpress)

Please outline where this information can be found within the submission (e.g., sections or figure legends), or explain why this information doesn’t apply to your submission:

The experiments quantifying microglial mobility (Fig 1C) was performed 5 times.

The experiments examining microglial interactions with the neuropil was performed 4 times.

The experiments examining real-time increases in microglial green fluorescence in the presence of sparsely labeled pHtdGFP axons (Fig 2E and Fig 2—fig supp 1) was performed 3 times.

The experiments quantifying microglial trogocytosis of pHtdGFP-labeled axons (Fig 3C-D, Fig 3—fig supp 1, and Fig 3—fig supp 2A) and SYP-pHtdGFP-labeled axons (Fig 3F and Figure 3—fig supp 2B) were performed 3 times.

The experiment quantifying microglial depletion with PLX5622 (Fig 4B-C) was performed once.
The experiment quantifying the effect of microglial depletion on retinal ganglion cell axon morphology (Fig 4E-F) was performed twice.

The experiment examining the effect of microglial depletion on animal behaviour was performed once (Fig 5D-K and Figure 5—fig supp 1).

The experiment examining the effect of aRCA3 overexpression on microglial trogocytosis (Fig 7D-7E and Fig 7—fig supp 1) was performed 5 times.

The experiment examining the effect of aRCA3 overexpression on axon morphology (Fig 7G-H) was performed 4 times.

The experiment examining the effect of VAMP2-C3 expression on axon morphology (Fig 8C-D) was performed 3 times.

The number of biological replicates (“n”) are reported in the results and figure legends. For retinal ganglion cell axon morphology experiments, a biological replicate is defined as an axon that remained discernible from other axons throughout the imaging session. In all other experiments, each tadpole was a biological replicate. In behavior studies, 10 trials (technical replicate) were performed on each tadpole. In microglia fluorescence studies, each microglial cell in the z-stack was a technical replicate. Data exclusion criteria is stated in the methods section. No outliers were excluded.

**Statistical reporting**

- Statistical analysis methods should be described and justified
- Raw data should be presented in figures whenever informative to do so (typically when N per group is less than 10)
- For each experiment, you should identify the statistical tests used, exact values of N, definitions of center, methods of multiple test correction, and dispersion and precision measures (e.g., mean, median, SD, SEM, confidence intervals; and, for the major substantive results, a measure of effect size (e.g., Pearson's r, Cohen's d)
- Report exact p-values wherever possible alongside the summary statistics and 95% confidence intervals. These should be reported for all key questions and not only when the p-value is less than 0.05.

Please outline where this information can be found within the submission (e.g., sections or figure legends), or explain why this information doesn’t apply to your submission:

Statistical analyses methods are described in figure legends, and further summarized in the Materials and Methods section.

(For large datasets, or papers with a very large number of statistical tests, you may upload a single table file with tests, Ns, etc., with reference to sections in the manuscript.)

**Group allocation**

- Indicate how samples were allocated into experimental groups (in the case of clinical studies, please specify allocation to treatment method); if randomization was used, please also state if restricted randomization was applied
- Indicate if masking was used during group allocation, data collection and/or data analysis

Please outline where this information can be found within the submission (e.g., sections or figure legends), or explain why this information doesn’t apply to your submission:

Groups were allocated based on treatment groups, such as vehicle control or drug-reared, or by electroporated plasmid construct. Masking was used during data analysis.

**Additional data files (“source data”)**

- We encourage you to upload relevant additional data files, such as numerical data that are represented as a graph in a figure, or as a summary table
- Where provided, these should be in the most useful format, and they can be uploaded as “Source data” files linked to a main figure or table
- Include model definition files including the full list of parameters used
- Include code used for data analysis (e.g., R, MatLab)
- Avoid stating that data files are “available upon request”

Please indicate the figures or tables for which source data files have been provided:

Source data for numerical data has been provided.

Code used for data analysis of tadpole behaviour can be found at www.github.com/tonykylim/XenLoom_beta
